# Correlation Between Intrafollicular IL-10, Progesterone, and Bovine Oocyte Developmental Competence

**DOI:** 10.3390/ijms262311364

**Published:** 2025-11-24

**Authors:** Aleksandra Teresa Pytel, Dawid Tobolski, Piotr Skup, Patrycja Strączyńska, Kinga Domrazek, Zdzisław Gajewski, Ewa Gorodkiewicz, Krzysztof Papis

**Affiliations:** 1 Department of Large Animal Diseases and Clinic, Institute of Veterinary Medicine, Warsaw University of Life Sciences, 02-776 Warsaw, Poland; aleksandra_zarzycka1@sggw.edu.pl; 2 Bovisvet Veterinary Practice of Reproduction and Cattle Diseases, 08-300 Kosierady Wielkie, Poland; piotr@bovisvet.com; 3 Gyncentrum Fertility Clinic, 31-332 Krakow, Poland; straczynska.patrycja@gmail.com; 4 Small Animal Reproduction Laboratory, Department of Small Animal Diseases with Clinic, Institute of Veterinary Medicine, Warsaw University of Life Science, Nowoursynowska 159, 02-776 Warsaw, Poland; kinga_domrazek@sggw.edu.pl; 5 Center of Translational Medicine, Warsaw University of Life Sciences, 02-797 Warsaw, Poland; 6 Bioanalysis Laboratory, Faculty of Chemistry, University of Bialystok, 15-245 Bialystok, Poland; 7 nOvum Fertility Clinic, 02-807 Warsaw, Poland

**Keywords:** bovine, oocyte competence, follicular fluid, interleukin-10, progesterone, SPRi biosensor, embryo development, IVP

## Abstract

The developmental competence of oocytes is a critical limiting factor in bovine in vitro embryo production (IVEP). Our study aimed to investigate the relationship between the intrafollicular concentrations of interleukin-10 (IL-10) and progesterone (P4), follicle characteristics, and the subsequent developmental success of bovine oocytes. Follicular fluid (FF) and corresponding cumulus–oocyte complexes (n = 314) were collected from FSH-stimulated heifers. A novel, high-sensitivity Surface Plasmon Resonance Imaging biosensor was used to quantify IL-10, while P4 was measured by an enzyme-linked fluorescent assay. Oocytes were individually cultured to assess cleavage (Day 3) and blastocyst formation (Day 7). Statistical analysis revealed that intrafollicular IL-10 concentration was a significant positive predictor of developmental success, significantly correlating with blastocyst rate (ρ = 0.29, *p* = 0.016). Oocytes from follicles with IL-10 concentrations above an optimized cutoff of 142.16 pg/mL had a 16.33-fold greater chance of developing into a blastocyst (*p* = 0.006). A predictive model combining IL-10 and oocyte morphology demonstrated the highest accuracy for predicting blastocyst success (AUC = 0.724). Conversely, poor oocyte morphology (Grade 4) and large follicular volume (>1200 µL) were significantly associated with developmental failure. Intrafollicular P4 concentration was not directly correlated with embryo development but rather with follicle size. Our findings identify intrafollicular IL-10 as a potent biomarker for predicting bovine oocyte competence and suggest that its quantification using sensitive biosensor technology could enhance the efficiency of IVEP programs.

## 1. Introduction

In vitro embryo production (IVEP) represents a fundamental technology in modern cattle breeding, with its application expanding at an average annual growth rate of 12%. The integration of IVEP with genomic selection and sexed semen has accelerated genetic gain, resulting in a quantity of in vitro-produced embryos surpassing those derived in vivo [1]. Despite these advances, the overall efficiency of bovine IVEP remains suboptimal. In vitro-produced embryos exhibit lower cryotolerance, and their transfer yields 10–40% lower pregnancy rates than those achieved with in vivo-derived counterparts. This reduced efficiency is largely attributed to a critical limiting factor: the variable and often insufficient developmental competence of the oocytes collected for the procedure [2]. Oocyte developmental competence (defined as the intrinsic ability of the gamete to complete meiosis, undergo successful fertilization, and subsequently develop to term) is acquired during a protracted period of folliculogenesis. The acquisition of competence is critically dependent on the molecular dialogue between the oocyte and its surrounding somatic cells within the ovarian follicle. The follicular fluid (FF), a complex ultrafiltrate of plasma progressively enriched with secretions from granulosa and theca cells, establishes the intrafollicular microenvironment. The follicular fluid contains a dynamic mixture of nutrients, growth factors, cytokines, and signaling molecules that collectively orchestrate oocyte growth, meiotic maturation, and the acquisition of embryonic potential [2]. Consequently, the composition of follicular fluid is the subject of intense investigation to identify reliable, non-invasive biomarkers that can predict oocyte quality and subsequent embryonic viability.

The biochemical characteristics of FF relate to the follicle development within an estrus cycle, which in turn corresponds with the early stages of oocyte meiosis resumption, as illustrated by the nucleolar chromatin condensation level, described as GV0 to GV3 stages [3]. Oocyte aspiration at random phases of the estrus cycle results in a mixture of GV1 to GV3 stages of immature oocytes. The GV1 oocytes have lower developmental competence than GV2 and GV3 [4]. Hormonal stimulation with FSH is commonly employed to maximize the number of preferred for reproduction GV2-stage oocytes. It should be noted that FSH administration increases follicle size; however, achieving proper chromatin condensation level requires the implementation of a specific coasting time (coasting period, CT), which corresponds to the plateau phase in the natural cycle prior to the LH surge [5].

Among the myriad of molecules present in FF, steroid hormones and cytokines have attracted significant attention. Progesterone (P4), a key steroid hormone, is integral to normal ovarian physiology, contributing to oocyte maturation, ovulation, and early embryo development [6]. In cattle, the terminal differentiation of the preovulatory follicle is marked by a hormonal shift from an estradiol-dominant to a P4-dominant state following the LH surge [7], with P4 concentrations increasing significantly in the final hours before ovulation [8]. While this transition is a hallmark of follicular maturity, the direct link between the final P4 concentration in an individual follicle and the competence of its resident oocyte remains a subject of investigation, with conflicting results reported from human assisted reproductive technologies (ART). Several studies indicate a positive relationship; higher FF P4 concentrations have been associated with successful fertilization and cleavage [9], and with expected fertilization outcomes following intracytoplasmic sperm injection (ICSI) [10,11]. A meta-analysis further supports an association between FF P4 concentration and fertilization success [12]. In porcine models, FF P4 positively correlates with oocyte developmental potential, and its supplementation during in vitro maturation enhances embryonic development [13]. Conversely, other studies have found no significant correlation or even negative associations. For instance, excessively high P4 levels have been linked to abnormal (polypronuclear) fertilization, potentially due to oocyte post-maturity in humans [14]. Furthermore, a negative correlation has been observed between FF P4 levels and the number of oocytes retrieved, as well as with the follicular antioxidant capacity [15]. These conflicting results suggest that the role of P4 is complex and that its effects may be mediated by intricate intracellular signaling pathways involving both nuclear and membrane receptors, which are crucial for oocyte developmental competence [16].

The ovarian follicle is also a site of significant immunological activity, where a delicate balance between pro- and anti-inflammatory signals is crucial for normal function [17]. Ovulation is recognized as a regulated inflammatory-like process, characterized by an influx of leukocytes and a cascade of cytokines [7,8]. Interleukin-10 (IL-10) is a potent anti-inflammatory cytokine, primarily produced by monocytes, macrophages, and various T cell subsets, known for its role in modulating immune responses and maintaining tissue homeostasis by suppressing the synthesis of pro-inflammatory mediators [18]. In human ART, the role of IL-10 is complex; higher concentrations in FF have been linked to successful fertilization and blastocyst formation [10,19], suggesting that IL-10 may contribute to a favorable, immune-tolerant microenvironment for oocyte development. However, other studies have reported lower FF IL-10 levels in women who achieved pregnancy [20] or no significant association with IVF outcomes in patients with diminished ovarian reserve [21]. Furthermore, elevated IL-10 has been observed in pathological conditions such as ovarian hyperstimulation syndrome and in obese women, where it is associated with markers of lower oocyte quality [22,23]. It is hypothesized that IL-10 may play a dual, context-dependent role, potentially limiting collateral damage from inflammatory processes associated with ovulation and enhancing the function of natural killer (NK) cells [24,25]. Data on the role of IL-10 in bovine reproduction is scarce, though it has been detected in pre-ovulatory follicular fluid, but becomes undetectable in the hours immediately preceding ovulation [8]. The accurate measurement of cytokines, often present at very low concentrations (pg/mL), poses a significant analytical challenge. Conventional methods like ELISA may lack the required sensitivity, with reported values often falling below the limit of detection [8].

To address this critical gap in bovine reproductive biology, a sensitive analytical approach is required to accurately quantify low-abundance cytokines in individual follicular fluid samples and correlate these measurements with developmental outcomes. This study was therefore designed to employ a novel, highly sensitive Surface Plasmon Resonance Imaging (SPRi) biosensor for the quantification of IL-10, alongside an enzyme-linked fluorescent assay (ELFA) for progesterone, in FF samples matched to individual cumulus–oocyte complexes. SPRi biosensors are novel tools that are successfully used to determine various biologically active molecules in body fluids [26,27]. We tested several hypotheses by tracking the subsequent in vitro development of each oocyte to the blastocyst stage. We initially hypothesized that higher intrafollicular concentrations of the anti-inflammatory cytokine IL-10 would positively correlate with oocyte developmental competence, as reflected by increased cleavage and blastocyst rates. Secondly, we speculated that intrafollicular progesterone concentration would not directly predict an individual oocyte’s fate but would instead correlate with follicle maturity and systemic endocrine status. Finally, we hypothesized that a predictive model combining a key biochemical marker (IL-10) with oocyte morphological grade would provide superior accuracy in forecasting blastocyst success compared to either parameter alone. The overall objective was to investigate the relationships between these intrafollicular biomarkers, physical follicle/oocyte characteristics, and developmental success to identify potent predictors for bovine IVEP programs.

## 2. Results

A total of 314 COCs and their corresponding follicular fluids were collected and analyzed. Following morphological evaluation, the oocytes underwent IVEP procedures. Overall, a cleavage rate of 63.7% was recorded on Day 3, and a blastocyst formation rate of 29.6% was achieved by Day 7.

### 2.1. Oocyte and Follicle Characteristics and Their Impact on Developmental Competence

**Figure 1:**
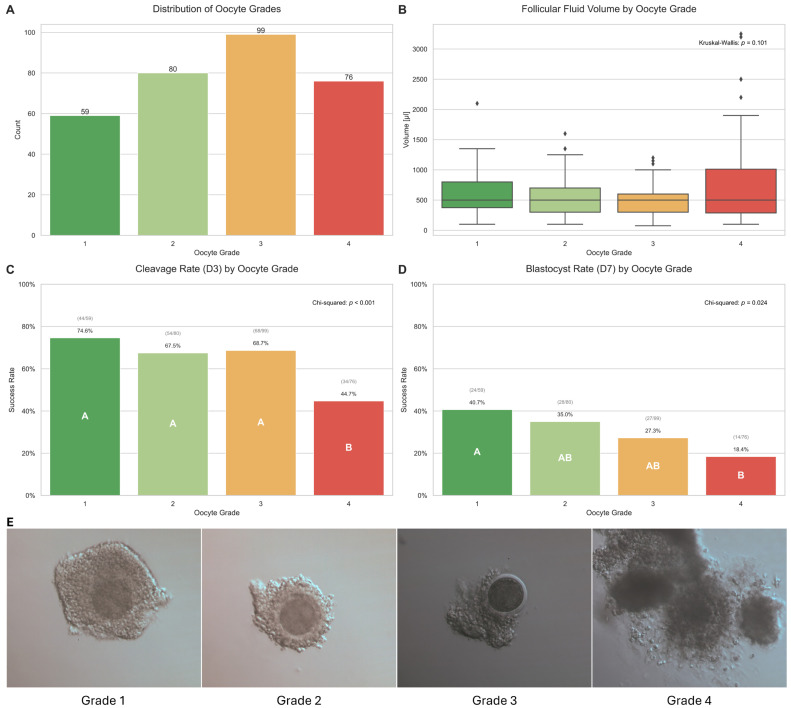
Oocyte and follicle characteristics in relation to developmental success. (**A**) Distribution of Oocyte Grades, showing the total count for each morphological category (n = 314). (**B**) Follicular Fluid Volume by Oocyte Grade, illustrating the range of volumes for each grade. (**C**) Cleavage Rate (D3) by Oocyte Grade, displaying the percentage of oocytes that successfully cleaved. (**D**) Blastocyst Rate (D7) by Oocyte Grade, showing the percentage of oocytes that developed to the blastocyst stage on Day 7. In panels (**C**,**D**), different letters (A, B) in the bars denote statistically significant differences between the grades (*p* < 0.05). (**E**) Representative bright-field microscopy images of oocytes classified according to morphological Grades 1–4.

The initial analysis focused on the baseline characteristics of the oocytes and follicles and their relationship with developmental success. The distribution of oocyte quality grades is presented in Figure 1A. The most frequent category was Grade 3 (n = 99; 31.5%), followed by Grade 2 (n = 80; 25.5%), Grade 4 (n = 76; 24.2%), and Grade 1 (n = 59; 18.8%).

While there was a trend towards slightly higher median fluid volumes for higher-quality oocytes, the analysis of follicular fluid volume did not reveal a statistically significant difference among the four oocyte quality grades (Kruskal–Wallis test: *p* = 0.101) (Figure 1B).

Crucially, the morphological quality of an oocyte was found to be a significant predictor of its developmental competence. A statistically significant decline in both cleavage rate (Day 3) and blastocyst formation rate (Day 7) was observed as oocyte quality decreased (Chi-squared test: *p* < 0.001 for D3 and *p* = 0.004 for D7). Oocytes classified as Grade 4 exhibited the lowest developmental potential, with a cleavage rate of only 44.7% and a blastocyst rate of 18.4% (Figure 1C,D). This was further confirmed by the odds ratio analysis, which showed that Grade 4 oocytes had significantly lower odds of reaching the cleavage stage (OR = 0.28, *p* = 0.001) and the blastocyst stage (OR = 0.34, *p* = 0.007) compared to Grade 1 oocytes (Table 1). Post-hoc analysis also confirmed that the developmental rates for Grade 4 oocytes were significantly lower than those for Grades 1, 2, and 3.

**Figure 2:**
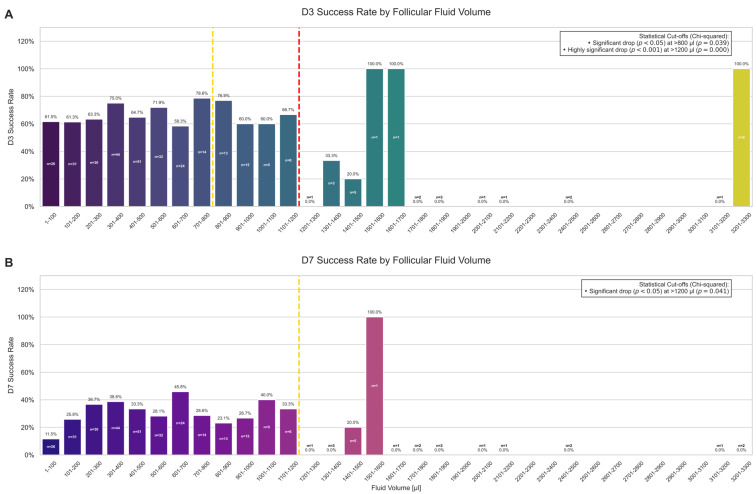
The effect of follicular fluid volume on embryo development rates. (**A**) The cleavage rate (D3) as a function of follicular fluid volume, grouped in 100 µL bins. A significant drop in success was identified for follicles with volumes greater than 800 µL (gold line, *p* <0.05) and a highly significant drop for volumes greater than 1200 µL (red line, *p* < 0.001). (**B**) The blastocyst rate (D7) as a function of follicular fluid volume. A significant decrease in blastocyst formation was observed for follicles with volumes exceeding 1200 µL (gold line, *p* < 0.05). Bar labels indicate the success rate and the number of oocytes (n) in each bin.

Furthermore, a higher follicular fluid volume was negatively associated with embryo development. A cutoff analysis identified volume thresholds beyond which developmental success rates significantly decreased. For the cleavage rate (D3), a significant drop was observed for follicles with a volume > 800 µL (*p* = 0.039), which became highly significant at >1200 µL (*p* < 0.001) (Figure 2A). A similar, though slightly less pronounced, relationship was found for the blastocyst rate (D7), where a statistically significant threshold was identified at >1200 µL (*p* = 0.041) (Figure 2B).

### 2.2. Relationships Between Biomarker Concentrations, Follicle Traits, and Embryo Development

**Figure 3:**
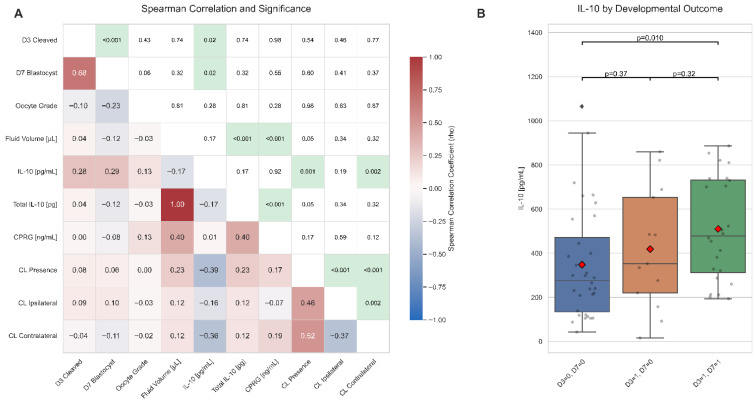
Correlation analysis of intrafollicular factors and their relationship with developmental outcome. (**A**) Heatmap of Spearman’s rank correlations. The lower triangle displays the correlation coefficients (ρ), while the upper triangle shows the corresponding *p*-values. Cells with statistically significant correlations (*p* < 0.05) in the upper triangle are highlighted with a green background. (**B**) Boxplot of intrafollicular IL-10 concentration grouped by developmental outcome: oocytes that failed to cleave (D3 = 0, D7 = 0), those that cleaved but did not form a blastocyst (D3 = 1, D7 = 0), and those that successfully developed to the blastocyst stage (D3 = 1, D7 = 1). Brackets indicate statistical differences between groups based on pairwise Mann–Whitney U tests.

Spearman’s rank correlation analysis was performed to investigate the inter-relationships between the measured variables (Figure 3A). These analyses were conducted on a subset comprising 69 COCs and their matched FF samples. The intrafollicular concentration of IL-10 (pg/mL) showed a significant, positive correlation with both the cleavage rate (ρ = 0.28, *p* = 0.019) and the blastocyst rate (ρ = 0.29, *p* = 0.016). In contrast, oocyte grade was negatively correlated with developmental success at both endpoints (D3: ρ = −0.10, *p* = 0.434; D7: ρ = −0.18, *p* = 0.060). The concentration of progesterone (CPRG, ng/mL) did not correlate with developmental outcomes (D3 cleavage: ρ = 0.003, *p* = 0.979; D7 blastocyst: ρ = −0.075, *p* = 0.549). CPRG correlated positively with follicular fluid volume (ρ = 0.405, *p* < 0.001), whereas its association with the presence of a corpus luteum (CL) was weak and not significant (overall CL presence: ρ = 0.173, *p* = 0.165; ipsilateral CL: ρ = −0.068, *p* = 0.585; contralateral CL: ρ = 0.194, *p* = 0.118).

These correlation findings were further substantiated when comparing IL-10 concentrations across groups with different developmental outcomes (Figure 3B). The median IL-10 concentration was significantly higher in the group of oocytes that successfully developed to the blastocyst stage (D3 = 1, D7 = 1) compared to those that failed to cleave (D3 = 0, D7 = 0) (Mann–Whitney U test: *p* = 0.01).

### 2.3. Predictive Value of IL-10 for Oocyte Developmental Competence

**Figure 4:**
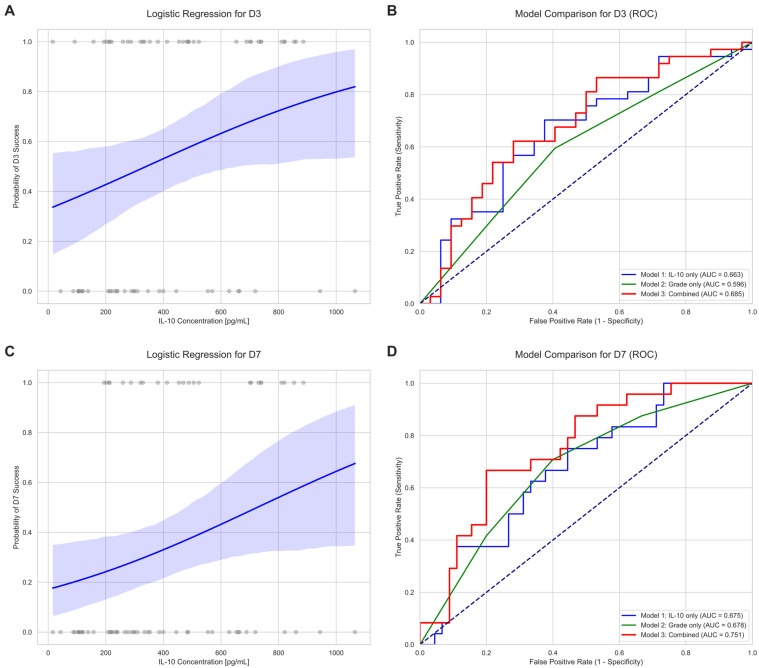
Predictive modeling of developmental success using intrafollicular IL-10 concentration and oocyte grade. (**A**) Logistic regression curve illustrating the positive relationship between IL-10 concentration and the probability of cleavage (D3). (**B**) Receiver Operating Characteristic (ROC) curves comparing the predictive performance for D3 success of three models: Model 1 (IL-10 only), Model 2 (oocyte grade only), and Model 3 (combined). (**C**) Logistic regression curve for the probability of blastocyst formation (D7). (**D**) ROC curves comparing the predictive models for D7 success, showing the superior performance of the combined model (AUC = 0.751) over the individual IL-10 (AUC = 0.675) and oocyte grade models (AUC = 0.678).

To formally assess the predictive capacity of IL-10, logistic regression models and Receiver Operating Characteristic (ROC) curve analysis were employed (Figure 4). Logistic regression confirmed that IL-10 concentration is a significant positive predictor for cleavage by Day 3 (coef. = 0.0021, *p* = 0.041) and blastocyst formation by Day 7 (coef. = 0.0022, *p* = 0.034) (Figure 4A,C, Table A1).

A comparison of predictive models using ROC analysis showed that a model based solely on IL-10 concentration (Model 1) had better discriminatory power (AUC for D3 = 0.663; AUC for D7 = 0.675) than a model based only on the oocyte’s morphological grade (Model 2) (AUC for D3 = 0.596; AUC for D7 = 0.678). Importantly, combining both IL-10 concentration and oocyte grade into a single model (Model 3) further enhanced its predictive power, especially for blastocyst development, yielding the highest Area Under the Curve (AUC for D7 = 0.751) (Figure 4B,D, Table A1). This combined model was also identified as the best-fitting model for predicting D7 success based on the Akaike Information Criterion (AIC = 87.37, Table A2).

Finally, an odds ratio analysis identified an optimal cutoff value for IL-10 at 142.16 pg/mL. Oocytes from follicles with IL-10 concentrations above this threshold had a 5.74-fold greater chance of cleavage by Day 3 (95% CI: 1.30–25.41; *p* = 0.018) and a remarkable 16.33-fold greater chance of developing to the blastocyst stage by Day 7 (95% CI: 0.92–290.51; *p* = 0.006) compared to oocytes from follicles with lower IL-10 levels (Table 1).

## 3. Discussion

The present study provides a comprehensive analysis of the intrafollicular concentrations of IL-10 and progesterone in relation to bovine oocyte developmental competence, uniquely enabled by applying a highly sensitive SPRi biosensor for IL-10 quantification. Our key finding that higher intrafollicular IL-10 concentration can be a potent positive predictor of blastocyst formation confirms our primary hypothesis and provides a critical new insight into the bovine follicular microenvironment. This result aligns with some investigations in human ART [10,19] and supports the concept that IL-10 is crucial for creating a balanced microenvironment that protects the maturing oocyte. As ovulation is an inflammatory-like process [7,8], the anti-inflammatory properties of IL-10 may be instrumental in shielding the oocyte from excessive inflammatory stress. IL-10 is characterized as a “late” cytokine, appearing after pro-inflammatory mediators, and its physiological role is to limit the immune response and prevent collateral tissue damage [25]. This protective function is likely critical for the oocyte during the inflammatory cascade of ovulation. It should also be emphasized that our outcome was defined as the attainment of the blastocyst stage by Day 7, and we did not apply a standardized morphological grading system to the resulting embryos. Consequently, the present data do not allow us to determine whether intrafollicular IL-10 is associated with blastocyst quality in addition to blastocyst formation, which represents an important direction for future work. However, the role of IL-10 in reproductive success is evidently complex and context-dependent. In contrast to our results, several human studies have associated high IL-10 levels with negative outcomes, particularly in pathological states like ovarian hyperstimulation syndrome [22] and obesity [23], or have reported lower IL-10 levels in women who achieved pregnancy [20]. In our study, only clinically healthy heifers with a normal body condition score (BCS) were used. Further research could include animals with higher BCS or with ovarian cysts to compare their IL-10 FF level. These discrepancies suggest that while elevated IL-10 in healthy, stimulated follicles may reflect a well-regulated anti-inflammatory state, it might represent a compensatory but ineffective response to underlying inflammation in certain human pathological conditions. The ability of our SPRi biosensor to accurately detect IL-10 at low pg/mL concentrations was critical, as subtle but significant variations may have been missed by less sensitive conventional assays, potentially explaining the scarcity of conclusive data in this area [28].

Consistent with our second hypothesis, the role of progesterone appears more complex and indirect. Although intrafollicular P4 concentration increased with follicle size, we found no direct, significant link to the developmental outcome of the resident oocyte. This result is consistent with recent bovine studies where no differences in blastocyst rates were observed between oocytes from follicles with high versus low intrafollicular P4, even though the high-P4 environment has altered gene expression in follicular cells [27]. The lack of a direct correlation implies that while a certain P4 threshold is necessary to indicate follicular maturity, excessively high concentrations do not confer an additional benefit and may not be a reliable standalone predictive marker.

The lack of a significant correlation observed between P4 and the presence of a corpus luteum (ρ = 0.17) further complicates its utility as a specific biomarker. This finding suggests that, in our dataset, the intrafollicular P4 concentration may not even strongly reflect the systemic endocrine status or local diffusion from the CL, reinforcing the conclusion that it is not a reliable standalone marker for the oocyte’s immediate microenvironment. While this contrasts with some reports indicating that follicles in an ovary ipsilateral to a CL have significantly higher P4 concentrations [29], it underscores the complex and perhaps variable nature of this relationship. Furthermore, the biological action of P4 is contingent on the presence and activity of its receptors (nPR, mPRs, PGRMC1/2) within the cumulus–oocyte complex [16,30], which may be a more critical determinant of competence than the absolute concentration of the hormone itself.

Our data also confirmed established predictors of oocyte quality while revealing new insights into follicular dynamics. It is already known that there is a correlation between oocyte morphology reflecting chromatin compaction level at the germinal vesicle (GV) oocyte stage, and that all GV stages can be found in all follicle sizes [31]. The GV1 oocytes are in a growing follicle, GV2 in the plateau stage, and GV3 in the early atresia stage or right before ovulation [3,4,32]. The significant negative correlation between poor oocyte morphology (Grade 4) and developmental success aligns with standard embryological assessment and validates the baseline parameters of our study. The newest research with the use of artificial intelligence also confirmed the importance of oocyte morphology—the oocytes with more than five compact layers of cumulus cells have a higher blastocyst rate, making it a more critical parameter than ooplasm appearance [33]. More revealing was the negative association between high follicular fluid volume and embryo development, with a significant decline in blastocyst rates observed in follicles larger than 1200 µL.

This finding suggests that in hormonally stimulated cycles, the largest follicles may not always harbor the most competent oocytes. This could be due to post-maturity or the onset of atresia, conditions known to compromise oocyte quality [2]. This is supported by findings where oocytes from follicles that have initiated atresia, as indicated by elevated follicular fluid androstenedione, fail to fertilize despite having a morphologically mature appearance [9]. In our study, follicles with higher volumes may have been on the verge of atresia, a state as—sociated with increased expression of apoptotic markers like CASP3, which has been linked to poor developmental outcomes [34].

From an applied perspective, IL-10 should be considered in the context of other follicular and systemic biomarkers already in use in cattle. Anti-Müllerian hormone (AMH), for example, is widely used as an indicator of ovarian reserve and oocyte quantity at the animal level, whereas IL-10, as assessed here, reflects the microenvironment and competence of individual oocytes. Future studies directly comparing the predictive performance of IL-10 alone and in combination with AMH could define whether these markers provide complementary or overlapping information for the design of in vitro embryo production programs [35,36].

Finally, we did not observe a significant correlation between higher intrafollicular IL-10 and morphological oocyte grade (ρ = 0.13). This indicates that the predictive value of IL-10 is directly linked to the underlying physiological processes of developmental competence, rather than the static morphological features visible at the time of collection. While IL-10 did not correlate with the visual grade, its strong association with successful development (Figure 3A, Table 1) suggests its protective role occurs at a molecular level, preserving essential functions not captured by standard morphological assessment. The molecular mechanisms of IL-10 action still offer a plausible link to oocyte biology. IL-10 signaling involves the activation of STAT transcription factors and can influence MAPK pathways [25,37]. Specific phosphatases regulated by IL-10, such as DUSP1/MKP1, are known to deactivate p38 MAPK, a kinase that plays a role in regulating meiotic arrest in oocytes [38,39]. This suggests a potential molecular pathway through which IL-10 could directly contribute to oocyte quality by modulating key signaling events involved in meiotic progression, thereby reinforcing its status as a significant biomarker for developmental competence. The intrafollicular environment is a complex biochemical milieu, and oocyte competence is ultimately determined by the interplay of numerous factors, including other cytokines, steroids, metabolites, and growth factors [7,34,40]. Our study identifies IL-10 as a key beneficial component of this environment in healthy, stimulated bovine follicles.

## 4. Materials and Methods

### 4.1. Ethical Statement

This study was conducted on donor heifers maintained under standard commercial dairy farm conditions. The animals were housed in free-stall barns with constant ad libitum access to fresh water and were fed a Total Mixed Ration (TMR) formulated to meet their nutritional requirements for growth and maintenance. All biological materials were collected during routine, non-experimental ovum pick-up (OPU) procedures performed within a commercial in vitro embryo production (IVEP) program. Because the sample collection was integrated into standard veterinary services and did not involve additional experimental interventions, formal ethical review and approval were not required under the Polish Act of 15 January 2015 on the Protection of Animals Used for Scientific or Educational Purposes, which implements the EU Directive 2010/63/EU.

### 4.2. Animal Stimulation and Follicular Fluid/Oocyte Collection

Follicular fluid (FF) and cumulus–oocyte complexes (COCs) were obtained from Holstein-Friesian heifers (n = 21). The animals were subjected to a controlled ovarian stimulation protocol consisting of five doses of follicle-stimulating hormone (FSH; Pluset, Calier, Barcelona, Spain). A defined coasting time (CT), representing the interval between the final FSH injection and OPU, was recorded for each session. Ovarian follicles were aspirated individually using an ultrasound-guided transvaginal procedure with an ultrasound scanner (BLUE ultrasound scanner, Draminski, Olsztyn, Poland). The aspiration system was connected to a 15-mL conical tube (Regulated Vacuum Pump, William A Cook Australia Pty Ltd., Queensland, Australia). After the aspiration of a single follicle, the tube was replaced, and the system was flushed with OPU collection medium (IVF Bioscience, Cornwall, UK) to prevent cross-contamination. The volume of the fluid from each follicle was measured. The corresponding oocyte was located within the collected fluid under a stereomicroscope (Olympus SZX-7, Olympus Corporation, Tokyo, Japan). Only fluids of which a COC was successfully retrieved were used for subsequent analysis. The dataset included follicular fluid volume and oocyte grade (according to the International Embryo Technology Society (IETS) classification) [41] for all 314 samples, as well as information on the presence of a corpus luteum on the ovary from which the follicle was aspirated for 69 samples.

### 4.3. In Vitro Embryo Production (IVEP)

A total of 314 COCs were processed. Immediately after retrieval, each COC was morphologically evaluated and graded from 1 to 4 according to the IETS classification system. The grading was based on a visual assessment of the number of cumulus cell layers, their degree of compaction, and the appearance of the ooplasm. Oocytes of the highest quality, designated as Grade 1, were characterized by having more than three complete and compact layers of cumulus cells and an ooplasm that appeared dense with even granulation. Grade 2 oocytes possessed fewer, specifically one or two, compact layers of cumulus cells while still maintaining a dense ooplasm with even granulation. It was also a criterion that oocytes meeting Grade 1 cumulus standards but exhibiting a speckled ooplasm were downgraded to Grade 2. A further reduction in quality led to a Grade 3 classification, which was assigned to oocytes with less than one complete layer of cumulus cells covering the zona pellucida. Finally, Grade 4 oocytes were identified by expanded cumulus cells, often with an agglutinated appearance. Any oocyte with a retracted ooplasm was considered degenerated. Beyond this scale, COCs were classified as degenerated if they presented with features such as a cracked zona pellucida, fused cumulus cells, or ooplasm that was retracted, speckled, or abnormally light [41]. The COCs were washed and placed individually into 50 µL drops of in vitro maturation (IVM) medium for 24 h. Subsequently, oocytes were individually fertilized for 10 h and, following denudation of cumulus cells, cultured individually in 50 µL drops of in vitro culture (IVC) medium on multi-well dishes (Birr BioSciences b.v., Vreeland, The Netherlands). Embryonic development was assessed on Day 3 post-fertilization for cleavage and Day 7 for blastocyst formation.

### 4.4. Sample Processing and Biomarker Analysis

Immediately after oocyte retrieval, the corresponding FF sample was placed in a 2 mL tube (Googlab Scientific Sp. z o.o., Rokocin, Poland) and centrifuged at 3000× *g* for 10 min at 4 °C (Eppendorf SE, Hamburg, Germany). The cell-free supernatant was transferred to a cryovial (Biologix Group Limited, Jinan, China) and stored in the vapor phase of liquid nitrogen (range between −150 °C to −196 °C) until analysis.

The concentration of IL-10 in FF samples (n = 69) was quantified using a novel, label-free SPRi biosensor, the development and validation of which have been described recently [42]. The assay is based on a direct immunosensing format where a specific polyclonal rabbit antibody is covalently immobilized on a gold sensor chip at a concentration of 10 µg/mL (pH 7.4). A small aliquot (3 µL) of the follicular fluid sample, diluted twofold with PBS, is applied to the functionalized surface. The specific binding between the immobilized antibody and IL-10 present in the sample causes an increase in mass at the sensor surface. This mass change alters the local refractive index, which the SPRi instrument monitors in real time as a change in the intensity of reflected light at a fixed angle. The magnitude of this change is directly proportional to the concentration of IL-10 in the sample, within a diagnostic range (1–1000 pg/mL). This method was selected for its high sensitivity (LOQ = 1.49 pg/mL) and minimal sample volume requirement, making it ideal for analyzing individual, volume-limited follicular fluid samples.

The concentration of progesterone in FF samples (n = 66) was determined using the automated VIDAS^®^ Progesterone (PRG) assay (bioMérieux, Marcy-l’Étoile, France) on a MINI VIDAS immunoassay analyzer. The assay operates on the enzyme-linked fluorescent assay (ELFA) competitive immunoassay principle. In this format, progesterone present in the sample competes with an alkaline phosphatase-labeled progesterone derivative (conjugate) for a limited number of binding sites on monoclonal anti-progesterone antibodies coating the interior of a Solid Phase Receptacle. Following the manufacturer’s instructions, a 200 µL aliquot of each follicular fluid sample was introduced into the ready-to-use reagent strip. The instrument performs all subsequent steps automatically, including sample incubation, washing steps to remove unbound components, and the final fluorescent detection step. The intensity of the fluorescent signal is inversely proportional to the concentration of progesterone in the sample. The entire process is completed within approximately 45 min. The assay has a validated measurement range of 0.25–80 ng/mL.

### 4.5. Statistical Analysis

All statistical analyses were performed using Python (Version 3.11) with SciPy 1.16, statsmodels 0.14, and scikit-learn 1.7 libraries for statistical computation, as well as Matplotlib 3.10.7 and Seaborn 0.13 for data visualization. A *p*-value < 0.05 was considered statistically significant for all analyses.

Data distribution was first assessed for normality using the Shapiro–Wilk test. Due to the non-normal distribution of key continuous variables such as IL-10 and progesterone concentrations, non-parametric tests were employed. The Mann–Whitney U test was used for comparisons between two independent groups (e.g., blastocyst vs. no blastocyst), while the Kruskal–Wallis test, followed by pairwise post-hoc tests with Bonferroni correction, was employed for comparisons across more than two groups (e.g., oocyte grades).

The relationship between categorical variables, such as oocyte quality grade and developmental outcomes (cleavage and blastocyst rates), was analyzed using the Chi-squared (χ^2^) test of independence. Fisher’s exact test was used for 2 × 2 contingency tables or when expected cell counts were low. Spearman’s rank correlation coefficient (ρ) was calculated to assess the monotonic relationship between ordinal and continuous variables.

Binary logistic regression models were constructed to evaluate the predictive value of intrafollicular IL-10 and oocyte grade on the binary outcomes of cleavage and blastocyst formation. The results are presented as odds ratios (OR) with corresponding 95% confidence intervals (CI). Three distinct models were compared for each outcome: Model 1 (IL-10 concentration as the sole predictor), Model 2 (oocyte grade as the sole predictor), and Model 3 (a combined model including both IL-10 and oocyte grade). The performance of these models was evaluated using Receiver Operating Characteristic (ROC) curve analysis, and the Area Under the Curve (AUC) was used as a measure of discriminatory power. The Akaike Information Criterion (AIC) was used to assess the relative goodness of fit among the models. An optimal cutoff value for IL-10 concentration was determined by iterative Fisher’s exact tests across various percentile-based thresholds to maximize the odds ratio for predicting developmental success.

## 5. Conclusions

The present study identifies intrafollicular IL-10 as a potent and positive predictive biomarker for bovine oocyte developmental competence. In contrast to progesterone, which did not directly correlate with embryo success, higher concentrations of IL-10 were significantly associated with an increased likelihood of development to the blastocyst stage. Using a highly sensitive SPRi biosensor was critical for this quantification and, when combined with morphological assessment, provided the most accurate predictive model for blastocyst success. These findings suggest that quantifying intrafollicular IL-10 could become a valuable tool for selecting oocytes with the highest developmental potential, thereby enhancing the efficiency of IVEP programs.

## Figures and Tables

**Table 1:** Impacts of Physiological, Biochemical, and Morphological Factors on Embryo Development at Day 3 and Day 7.

Predictor Group	Predictor/Comparison	Outcome	Odds Ratio (95% CI)	*p*-Value
Physiological Factors *	CL Presence (Yes vs. No)	D3	1.40 (0.48–4.10)	0.584
		D7	1.32 (0.42–4.17)	0.771
	Ipsilateral CL (Yes vs. No)	D3	1.44 (0.55–3.75)	0.471
		D7	1.52 (0.57–4.10)	0.446
	Contralateral CL (Yes vs. No)	D3	0.87 (0.34–2.21)	0.812
		D7	0.64 (0.24–1.73)	0.449
Biochemical Marker *	IL-10 > 142.16 pg/mL	D3	5.74 (1.30–25.41)	**0.018**
	IL-10 > 142.16 pg/mL	D7	16.33 (0.92–290.51)	**0.006**
Morphological Factor *	Oocyte Grade (vs. Grade 1)			
	Grade 2	D3	0.72 (0.34–1.50)	0.452
		D7	0.79 (0.40–1.57)	0.595
	Grade 3	D3	0.76 (0.37–1.55)	0.473
		D7	0.55 (0.28–1.08)	0.113
	Grade 4	D3	0.28 (0.14–0.59)	**0.001**
		D7	0.34 (0.16–0.73)	**0.007**

* Analyses for Physiological Factors and the Biochemical Marker were conducted on a subset of oocytes for which IL-10 data were available (n = 69). The Morphological Factor analysis was conducted on the larger dataset of oocytes with complete grade and outcome data (n = 314). CL: Corpus Luteum; CI: Confidence Interval. *p*-values were calculated using Fisher’s exact test. Significant *p*-values (*p* < 0.05) are shown in bold.

## Data Availability

The data presented in this study are available on request from the corresponding author.

## Appendix A

**Table A1:** Logistic regression models of the coefficients for predicting cleavage (D3) and blastocyst (D7) success. The table details the coefficients (β), 95% confidence intervals, and *p*-values for each predictor variable within the three logistic regression models evaluated for both developmental outcomes.

Outcome	Model	Predictor	Coefficient (β)	95% Confidence Interval (CI)	*p*-Value
D3 Success	Model 1	IL-10 [pg/mL]	0.0021	0.000–0.004	0.041
(Cleavage)	(IL-10 only)	Intercept	−0.711	−1.649–0.228	0.138
	Model 2	Oocyte Grade 2 (vs. 1)	−0.79	−2.148–0.568	0.254
	(Grade only)	Oocyte Grade 3 (vs. 1)	−0.028	−1.404–1.348	0.968
		Oocyte Grade 4 (vs. 1)	−0.762	−2.079–0.554	0.257
	Model 3	IL-10 [pg/mL]	0.0022	0.000–0.004	0.046
	(Combined)	Oocyte Grade 2 (vs. 1)	−0.863	−2.261–0.536	0.227
		Oocyte Grade 3 (vs. 1)	−0.481	−1.968–1.007	0.526
		Oocyte Grade 4 (vs. 1)	−0.889	−2.254–0.476	0.202
D7 Success	Model 1	IL-10 [pg/mL]	0.0022	0.000–0.004	0.034
(Blastocyst)	(IL-10 only)	Intercept	−1.575	−2.616–(−0.533)	0.003
	Model 2	Oocyte Grade 2 (vs. 1)	−1.204	−2.650–0.242	0.103
	(Grade only)	Oocyte Grade 3 (vs. 1)	−0.357	−1.693–0.980	0.601
		Oocyte Grade 4 (vs. 1)	−1.715	−3.247–(−0.183)	0.028
	Model 3	IL-10 [pg/mL]	0.0025	0.000–0.005	0.03
	(Combined)	Oocyte Grade 2 (vs. 1)	−1.324	−2.831–0.182	0.085
		Oocyte Grade 3 (vs. 1)	−0.936	−2.445–0.573	0.224
		Oocyte Grade 4 (vs. 1)	−1.954	−3.575–(−0.333)	0.018

**Table A2:** A comparison of predictive model performance for cleavage (D3) and blastocyst (D7) success. The table presents key statistical metrics, including Log-Likelihood, Pseudo R^2^, Akaike Information Criterion (AIC), and Likelihood Ratio Test (LLR) *p*-value, to compare the goodness-of-fit and predictive accuracy of the three models for each outcome.

Outcome	Model	Predictors	Log-Likelihood	Pseudo R^2^	AIC	LLR *p*-Value
D3 Success	Model 1	IL-10 only	−45.384	0.047	94.77	0.033
	Model 2	Grade only	−46.419	0.026	100.84	0.484
	Model 3	Combined	−44.297	0.07	98.59	0.153
D7 Success	Model 1	IL-10 only	−42.21	0.053	88.42	0.029
	Model 2	Grade only	−41.216	0.075	90.43	0.081
	Model 3	Combined	−38.685	0.132	87.37	0.019
